# Modeling pathogen-driven neonatal late-onset sepsis: a modification to the murine cecal slurry

**DOI:** 10.3389/fcimb.2025.1589712

**Published:** 2025-06-10

**Authors:** Camryn Sellers-Porter, Shiloh R. Lueschow-Guijosa, Jessica M. Santana, Anjali J. Cera, Geoanna M. Bautista, Michele Persiani, Misty Good, Steven J. McElroy

**Affiliations:** ^1^ Department of Pediatrics, University of California, Davis, Davis, CA, United States; ^2^ Department of Pediatrics, University of Iowa Health Care, Iowa City, IA, United States; ^3^ Department of Pediatrics, University of North Carolina Hospitals, Chapel Hill, NC, United States

**Keywords:** neonatal sepsis (NS), macrophage - cell, monocyte - macrophage, necrotizing enterocolitis (NEC), murine (mouse), microbiome

## Abstract

**Introduction:**

Neonatal sepsis is a major cause of neonatal morbidity and mortality. Reliable animal models are essential to our understanding of late-onset sepsis, but notable limitations exist in the current standard murine cecal slurry model. We sought to refine the existing model by using an injection of known stock slurry (“NEC’teria”) cultured from an infant who died of necrotizing enterocolitis to better mimic sepsis following the translocation of neonatal specific bacterial pathogens from the intestine into the peritoneum.

**Methods:**

To induce sepsis, neonatal mice (P7 and P14 – P16) were given an intraperitoneal injection of varying concentrations of NEC’teria, while sham controls received an injection of PBS. Mice were monitored for survival and tissue samples, serum, and peritoneal washes were collected for further assessment of inflammation, immune response, and intestinal injury. Ceca were collected for microbiome analysis.

**Results:**

While the polymicrobial cecal slurry from adult mice contained common healthy gut microbes, NEC’teria is composed of bacteria, primarily from the Enterobacteriaceae and Enterococcaceae families, that are common causes of late-onset sepsis. NEC’teria exposure significantly increased serum inflammatory cytokines, resulted in intestinal injury, altered the microbiome composition, and induced significant changes in local and systemic immune cell expression. Sepsis-induced mortality, inflammation, and intestinal injury were live-bacteria dependent and could be attenuated by administration of an antibiotic one hour after bacterial injection.

**Discussion:**

Our modification to the cecal slurry neonatal sepsis model resulted in a consistent sepsis-related mortality and phenotypic changes in neonatal mouse pups that resembled the changes that occur in human preterm infants who develop late-onset sepsis. Our pathogenic slurry is highly relevant to neonatal sepsis, as it is comprised of bacterial families found commonly in septic neonates. We expect our model to be highly reproducible between institutions, due to the standardized bacterial dose and characterized stock solution.

## Introduction

Neonatal sepsis is a major cause of neonatal morbidity and mortality (Shane et al., 2017) (Stoll et al., 2015), remaining in the top ten causes of neonatal death in the United States (Ely and Driscoll, 2024) and top three causes worldwide (Oza et al., 2015) (Liu et al., 2016). Neonatal sepsis is a systemic response to infection or inflammation of typically sterile body fluids, that results in a potent release of pro-inflammatory cytokines and hemodynamic changes. Without rapid intervention, neonatal sepsis can ultimately result in multisystem organ failure and death (Fleiss et al., 2021) (Singer et al., 2016) (Tsai et al., 2014). Neonatal sepsis disproportionately impacts neonates that are born prematurely, affecting up to 30% of preterm infants, with incidences as high as 60% among cohorts of lower gestational age (Puopolo et al., 2018) (Greenberg et al., 2017) (Stoll et al., 2002) (Stoll et al., 2015). A driving cause of the increased incidence in preterm infants may be due to an immature gastrointestinal tract, characterized by a “leaky” or underdeveloped barrier function phenotype that allows bacteria to translocate from the lumen of the intestine into the bloodstream, leading to sepsis (Lin et al., 2008) (Carbonaro et al., 1988) (Chen et al., 2014) (Shulhan et al., 2017). The timing of onset of neonatal sepsis influences the probable etiology of infection. Early-onset sepsis (EOS) occurs within the first 72 hours of life due to pathogens acquired during the peripartum period, while late-onset sepsis (LOS) occurs after the first 72 hours of life due to pathogens acquired from the hospital or community setting (Glaser et al., 2021) (Shane et al., 2017). While infants who develop EOS are generally infected with bacteria such as *Group B Streptococcus* and *Escherichia coli* (*E. coli*) that are colonizers of the maternal genitourinary tract (Briggs-Steinberg and Roth, 2023), the pathogens associated with late-onset sepsis are instead commonly found in the intestine of preterm infants. These include gram-negative organisms in the Enterobacteriaceae family- such as *Escherichia coli* (*E. coli*), *Klebsiella* species, and *Enterobacter* species- and the gram-positive organisms *Staphylococcus aureus*, *Staphylococcus epidermidis* and *Enterococcus* species (Cailes et al., 2015) (Dong et al., 2019) (Stoll et al., 2002).

The “gold standard” murine model of adult sepsis is the cecal ligation and puncture (CLP) model. The CLP model mimics intestinal spillage into the peritoneum by puncturing the intestine following surgical ligation of the cecum, resulting in a polymicrobial sepsis phenotype that provides a reasonable surrogate for adult human sepsis (Dejager et al., 2011). However, translating this model into neonatal mice to mimic neonatal sepsis has been challenging as their small size, varying anesthesia needs, and anatomy make cecal ligation impractical (Rincon et al., 2021). This has limited most murine models of neonatal sepsis to those that can be induced through injection, such as an injection of a single strain of live bacteria or LPS (the toxemia model). While these methods allow for high consistency and reproducibility, they are less translatable to human polymicrobial sepsis than a polymicrobial bacterial infection model (Libert et al., 2019). Wynn and colleagues adapted the CLP model for neonatal mice by intraperitoneally injecting a fixed amount of cecal slurry contents harvested from an adult donor mouse into pups to induce sepsis (cecal slurry (CS) model) (Young et al., 2017) (Nolan et al., 2020) (Wynn et al., 2007). This model is now widely used, as it accurately recreates a polymicrobial sepsis and subsequent immune response. However, limitations remain in using the CS model for studying particular forms of neonatal sepsis commonly seen in clinical practice, such as those arising from necrotizing enterocolitis (NEC) or spontaneous intestinal perforations (SIP). NEC and SIP are both severe gastrointestinal conditions highly associated with prematurity. SIP is a non-inflammatory isolated perforation of the small bowel that is predominantly seen in the most premature infants in the first two weeks of life (Swanson et al., 2022). NEC is also predominantly seen in preterm infants but is an acute inflammatory process that occurs weeks after birth and is characterized by a significant increase in Enterobacteriaceae species shortly before onset (Tanner et al., 2015) (Claud and Walker, 2001) (Neu and Walker, 2011) (Mizrahi et al., 1965), and induces small bowel necrosis (Mara et al., 2018). Both conditions lead to perforation of the bowel followed by spillage of intestinal bacteria and contents into the peritoneal cavity and can cause subsequent sepsis (Speer et al., 2024). Additionally, LOS can occur in preterm infants without SIP or NEC due to the immature and leaky intestinal barrier at times of inflammation that may allow for translocation of bacteria from the preterm intestinal microbiome (Sharma et al., 2007). Since it is well known that the intestinal microbiome is different in preterm infants compared to children and adults (La Rosa et al., 2014), and inflammatory diseases such as NEC are associated with dysbiotic microbiomes (Morrow et al., 2013), a model that uses neonatal-specific bacteria that is known to be pathogenic and administered in a consistent and reproducible dose is needed to closer model neonatal LOS (Osuchowski et al., 2018). We therefore sought to refine the CS model by using an injection of known stock slurry cultured from an infant who died of necrotizing enterocolitis (“NEC’teria”) (Good et al., 2014) to better mimic sepsis following the translocation of neonatal specific bacterial pathogens from the intestine into the peritoneum. We provide our findings using this model regarding lethality, inflammatory and immune response, and effects on the microbiome.

## Methods

### Animals

C57Bl/6J mice were acquired from Jackson Laboratories (Bar Harbor, ME, USA) and then bred and raised at the University of Iowa according to protocols approved by the Institutional Animal Care and Use Committee (IACUC) (Protocol #8041401 and #0082328), and at the University of California, Davis according to protocols approved by the IACUC (protocol #24099). Postnatal day (P) 14–16 mice were used in the majority of our experiments as in terms of gene regulation, they are intestinally equivalent to a 22 – 24-week infant at the cusp of viability (Stanford et al., 2020). Validation of the sepsis model was also performed in P7 mice to ensure there were not significant age differences in the response. Neonates were housed with their mothers in the vivarium until the day of the experiment when they were moved to the laboratory space for closer monitoring.

P7 and P14 – 16 neonatal mice in the model establishment and validation experiments were kept with their mothers for the entire experiment and checked for survival at the 6, 12, 24, 36, 48, 60, 72, 96, and 120-hour time points. After establishment of the model and timing, P14 - P16 mice in the meropenem and experimental therapeutic groups were taken from their mothers and kept in temperature- and humidity-controlled chambers for the remainder of the experiment. Mice removed from their mother were given an oral gavage of Pedialyte (Abbott labs, Columbus, OH, USA) at the six-hour time point to maintain hydration through the remainder of the experiment. All mice were sexed and balanced between groups and litters were split up into the various experimental groups as well to prevent sex-specific or litter-specific confounding of the results. Euthanasia was performed in accordance with IACUC approval by using CO2 at 2.5L flow/min/chamber volume.

### Mouse cecal slurry for generalized peritonitis

The bacterial microbiome used in Wynn et al., 2007 as a model for polymicrobial sepsis was assessed (Wynn et al., 2007). In brief, seven-week-old female C57Bl/6 mice were obtained from Jackson Laboratories (Bar Harbor, ME, USA) in two separate shipments. Three mice were euthanized each week for the two trial weeks (cohort 1 and cohort 2) within twelve hours of arrival at the institution. The cecum of the mice was excised, and scissors were used to open the cecum at its most distal point. Forceps were used to squeeze the cecal contents into a 1.5 mL Eppendorf tube and one milliliter of five percent dextrose was added. The cecal slurry was vortexed briefly to homogenize and then was frozen at -20°C until ready for extraction.

### Pathologic slurry model

To understand the appropriate dose of NEC’teria needed to induce sepsis in neonatal mice, P7 or P14 – P16 mice (nine mice per group or fifteen mice per group, respectively) were given an intraperitoneal injection of varying concentrations of NEC’teria and monitored for survival through the remainder of the experiment at the times listed above. To understand if experiments were dependent on live bacteria, eight to ten P14 – P16 mice per group were injected intraperitoneally with 3 x 10^7^ CFU in the sepsis group as a positive control, while a separate group received an intraperitoneal injection of PBS and an intraperitoneal injection an hour later with 0.1 milligrams per kilogram body weight (mg/kgbw) Meropenem as a sham control. The experimental group received an intraperitoneal injection of 3 x 10^7^ CFU NEC’teria and an intraperitoneal injection an hour later with 0.1 mg/kgbw Meropenem. To further characterize the model, 12 hours following injection with NEC’teria, serum was harvested from 5 mice per group for analysis of cytokines, an ileal section was harvested to analyze intestinal injury on a generalized scale, and the cecum was harvested to analyze the microbiome.

### Serum cytokine measurements

Blood obtained via facial vein puncture was incubated on ice for one hour following collection and then centrifuged at 7000 RPM for five minutes to isolate the serum. Cytokines were then quantified using a Meso-Scale Discovery V-Plex Assay (Meso-Scale, Gaithersburg, MD, USA) according to manufacturer’s instructions. Plates were read on a Sector Imager 2400 at 620 nm. All samples were run in duplicate with the cytokines being read including IL-10, IL-6, KC-GRO, TNF, and IL-17A.

### Injury scoring

Ileal intestinal injury was performed by a single blinded investigator as previously described on 6 mice per group (Lueschow-Guijosa et al., 2024). In brief, Hematoxylin and Eosin (H&E) staining was performed on ileal sections and tissue was evaluated using a Nikon Eclipse NiU microscope (Nikon, Melville, NY, USA) for generalized injury on a three-point scoring scale (0 = normal, 1 = mild, 2 = severe) based on the degree of villus vacuolization, mucosal ulceration, lamina propria (LP) damage, and presence of hemorrhage within villi.

### Microbiome analysis

Cecal microbial analysis was performed as previously described (Lueschow-Guijosa et al., 2024). In brief, five neonatal ceca per group were removed from euthanized mice at the twelve-hour time point and stored at -80°C until processing. A ZymoBIOMICS Fecal/Soil DNA MiniPrep kit (Zymo Research, Irvine, CA) was used to extract DNA from the intact ceca of neonatal mice and the polymicrobial cecal slurry of adult mice. Extracted DNA was stored at -20°C until ready for further processing. Extracted DNA was quantified using the QuantIT dsDNA kit, High Sensitivity (Waltham, MA, USA).

For the neonatal cecum DNA, 16S rDNA amplification and sequencing were performed as previously described using the Earth Microbiome Project standard protocols (www.earthmicrobiome.org) using the V4 domain and the following primers: F515 (5’-NNNNNNNNGTGTGCCAFCMGCCGCCGCGGTAA-3’) and R806 (5’-GGACTACHVGGGTWTCTAAT-3’), with the forward primer modified to contain a unique 8 nucleotide linker sequence (italicized poly-N section of the primer above) and a 2-nucleotide linker sequence (bold, underlined portion) at the 5’ end. PCR reactions used 5–100 ng DNA template, 1X GoTaq Green Master Mix (Promega, Madison, WI), 1 mmol/L MgCl2, and 2 pmol of each primer. PCR was performed at 94°C for the initial 3 minutes followed by 35 cycles of 94°C for 45 s, 50°C for 60 s, and 72°C for 90 s, with a final extension of 72°C for 10 minutes. PCR amplicons were grouped at approximately equal amplification intensity ratios and were purified using the Qiaquick PCR purification kit (Qiagen, Hilden, Germany). The PCR amplicons were submitted to the UC Davis Genome Center DNA Technologies Core for Illumina paired-end library preparation, cluster generation, and 2x300 bp paired-end Illumina MiSeq sequencing.

For the adult cecal slurry DNA, 16S rDNA amplification and sequencing were performed as previously described (Shahi et al., 2019) using the V3-V4 domain and the following primers F (5’-TCGTCGGCAGCGTCAGATGTGTATAAGAGACAGCCTACGGGNGGCWGCAG-3’) R (5’-GTCTCGTGGGCTCGGAGATGTGTATAAGAGACAGGACTACHVGGGTATCTAATCC-3’). PCR reactions used 5–100 ng DNA template, KAPA HiFi HotStart ReadyMix (2X) (Kapa Biosystem, MA, USA), and 1 µL of each primer at 1 µM concentration. PCR was performed at 95°C for the initial 3 minutes followed by 25 cycles of 95°C for 30 s, 55°C for 30 s, and 72°C for 30 s, with a final extension of 72°C for 5 minutes. Index barcodes were added using a secondary PCR with thermocycler parameters of 95°C for 3 min followed by eight cycles of 95°C for 30 s, 55°C for 30 s, and 72°C for 30 s and a final extension at 72°C for 5 min. Amplicons were then cleaned using an Agencourt AMPure XP magnetic bead kit (Beckman Coulter, IN, USA) and quantified using a Bioanalyzer DNA 1000 chip (Agilent Technologies, CA, USA). PCR amplicons were grouped at approximately equal amplification intensity ratios and then submitted to Holden Comprehensive Cancer Center Microbiome Core at the University of Iowa for 2x300 bp paired-end Illumina MiSeq sequencing.

Data from the sequencing runs was analyzed using the QIIME software package (University of Colorado, Boulder, CO, version 2 2021.8.0). Sequences were demultiplexed and then DADA2 was used as a quality filter with default settings to exclude chimeric and low-quality sequences. This step also split sequences into 100% operational taxonomic units (OTUs)/amplicon sequence variants (ASVs). A secondary filtration was performed to remove low-abundance ASVs at a threshold of 0.005%. Filtered ASVs were taxonomically classified using the Greengenes 16S rRNA database (gg_13_8 release). Relative abundances of various taxa were acquired from combining the ASV classification with the frequency counts of the ASVs from the DADA2 step. EMPeror weighted UniFrac principal coordinate analyses (PCoAs) were generated to examine beta diversity.

### Minion oxford nanopore analysis

Cultures were pelleted at 5000 RPM for 10 minutes at room temperature and then a ZymoBIOMICS Fecal/Soil DNA MiniPrep kit (Zymo Research, Irvine, CA) was used to extract DNA. The DNA was then purified using a MasterPure Gram Positive DNA Purification Kit (Lucigen, Middleton, WI, USA) and then the DNA was stored at -20°C until it was ready for further processing. Sequencing libraries were prepared from high molecular weight gDNA using the SQK-RBK004 rapid chemistry kit (Oxford Nanopore Technologies, Oxford, UK) according to the manufacturer’s specifications. Samples were barcoded and the libraries were pooled to equimolar concentrations. Pooled samples were sequenced on the Oxford Nanopore MinION platform (Oxford Nanopore Technologies, Oxford, UK) using an R9.4.1 flow cell (Oxford Nanopore Technologies, Oxford, UK) at Evolve Biosystems laboratory (Davis, CA, USA).

Samples were demultiplexed and adapters were trimmed with qcat v1.1.0. Trimmed, demultiplexed reads were classified with Kraken2 using a pre-built database (minikraken2_v2_8GB_201904_UPDATE) built from Refseq’s bacterial, archaeal, and viral domains. Additionally, trimmed reads greater than or equal to 1000bp were assembled using canu v1.8 with the modified parameters optimized for metagenome assembly (maxInputCoverage=10000 corOutCoverage=10000 corMhapSensitivity=high corMinCoverage=0 redMemory=32 oeaMemory=32 batMemory=200). Both assembled contigs and unassembled reads were classified against the Refseq bacterial genome database using Blastn.

### Intraperitoneal wash and culture analysis

Eight to ten mice were euthanized at 10 hours following sepsis induction, with or without Meropenem treatment, or sham injection as described above. Following euthanasia, the abdominal cavity was lavaged with sterile PBS. Peritoneal fluid was then collected, plated on Luria agar plates, and incubated 37°C for 12 hours. Colony forming units (CFU) were manually counted.

### Flow cytometric analysis

Nine to twelve mice per group were exposed to sepsis with 3x10^7 CFU of NEC’teria or a saline control and sacrificed at 4 hours after injection. For splenic cell isolation, spleens were homogenized by manual dissociation and red blood cells were lysed in ACK- lysing buffer (Thermo-Fisher Scientific, Waltham, MA) for 5 min on ice. Remaining cells were washed and resuspended in 1 × PBS prior to staining. For single cell suspension of lamina propria (LP) leukocytes, small intestines were removed from mice and placed in ice cold PBS. Fat and fecal material was manually removed, intestines were segmented and longitudinally cut to expose the mucosal layer. Samples were then incubated in 2mM EDTA prior to repeated vortexing to remove the intraepithelial layer. Samples were incubated in digestion medium (2mg/ml collagenase I, 50ug/ml DNase I in RPMI containing 10% FBS), and washed with RPMI with 10% FBS. Samples were suspended in 40% Percoll in PBS, underlaid with 80% Percoll, and centrifuged for density gradient isolation of leukocytes. Cells were washed and resuspended in PBS with 10% FBS prior to staining for flow cytometry.

The following antibodies were used for staining: PE-Cy7 conjugated anti-mouse CD3, PE conjugated anti-mouse CD4, FITC conjugated anti-mouse CD8, PE conjugated anti-mouse CD11b, FITC conjugated anti-mouse CD11c, PE-efluor 610 conjugated anti-mouse CD25, APC conjugated anti-mouse Ly6C, PE-Cyanine5 conjugated anti-mouse Ly6G, Alexa Fluor 700 conjugated anti-mouse MHC Class II, Brillian Violet 605 conjugated anti-mouse NK1.1, unconjugated anti-mouse CD16/CD32 (all from eBioscience inc, Santa Clara, CA), APC-Cy7 conjugated anti-mouse CD19 (BD Biosciences, San Jose, CA), PE-Texas Red conjugated anti-mouse F4/80 (Invitrogen, Waltham, MA). Ultracomp eBeads plus (Invitrogen, Waltham, MA) were used for compensation controls. All samples were fixed in 1% paraformaldehyde before analysis.

Flow cytometry was run on a BD LSR II™ flow cytometer (BD Biosciences, San Jose California, USA) with the support of the UC Davis Flow Cytometry Shared Resource Center. Data was collected using BD FACSDiva™ software (BD Biosciences, San Jose California, USA). Data was analyzed using FlowJo Version 10 Software (FlowJo, Ashland, Oregon, USA).

### Statistical analysis/experimental design

ANOVA and non-parametric Kruskal–Wallis testing and t-tests were performed to determine statistical significance using GraphPad Prism v8. Significance was set as p < 0.05 for all experiments. All experiments were performed in at least triplicate and sample sizes were based on prior studies and published data. *In vivo* experimental groups were divided between litters and represent multiple experiments to prevent bias. Animal numbers used in all experiments are listed in the methods and figure legends.

## Results

### The bacteria present in NEC’teria are more common causes of LOS than bacteria in adult murine cecal slurries

**Figure 1:**
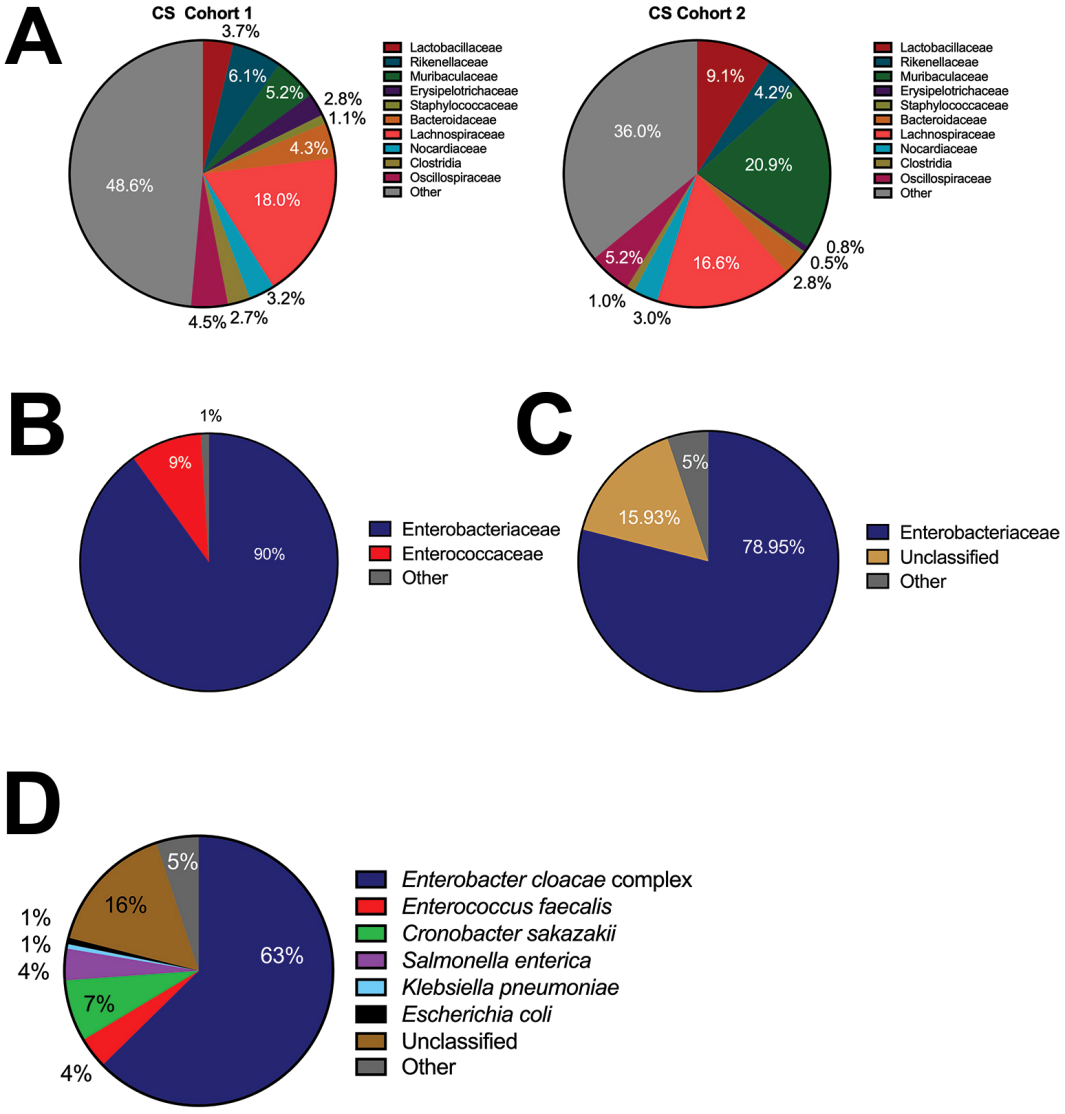
Bacterial composition of the commonly used cecal slurry compared to NEC’teria pathogenic slurry. To determine the composition of the polymicrobial cecal slurry and NEC’teria, we performed Illumina sequencing **(A)** with reported family level relative abundances of the cecal slurry of two cohorts of mice **(B)** and reported family level relative abundances of the NEC’teria. **(C)** MinION sequencing was performed to further assess the NEC’teria family level relative abundances, and **(D)** NEC’teria species level relative abundances. Percentage of each family **(A–C)** or species **(D)** identified are as listed.

Using Illumina MiSeq sequencing, we assessed the bacterial composition of the cecal slurry at two separate time points from two separate shipments. The cecal slurries obtained from adult C57Bl/6 female mice from Jackson Laboratories had high diversity in the families observed (**Figure 1A**). While the cecal slurries were relatively consistent between cohort 1 and cohort 2 there were significant differences in the Muribaculaceae family and the other category with cohort 1 having more bacteria categorized as other (48.6% vs 39.5%) and less categorized as Muribaculaceae (5.2% vs 20.9%) than cohort 2. In both cohorts, Lachnospiraceae, a common gut anaerobe, was detected in high abundance compared to the other bacteria. In contrast to the Wynn et al. model, our pathologic sepsis model used NEC’teria (Good et al., 2014) as the inciting element for LOS, however the exact makeup of NEC’teria has also not previously been fully defined in the literature. Illumina 2^nd^ generation sequencing was performed to provide a broad overview of the bacteria present in the culture. Results showed that 90% of the bacteria present were in the Enterobacteriaceae family (**Figure 1B**), which is consistent with our knowledge of NEC presentation. Another 9% were in the Enterococcaceae family, while the remaining 1% were in the unclassified or other category. To further characterize the NEC’teria and make species level distinctions, 3^rd^ generation sequencing was performed (**Figures 1C, D**). Results from this sequencing indicated 78.1% of bacteria were in the Enterobacteriaceae family, with only 3.6% in the Enterococcaceae family (**Figure 1C**). Within the Enterobacteriaceae family, the majority of total bacteria, ~61%, were within the *Enterobacter cloacae* complex, while 7.45% were *Cronobacter sakazakii*, 1.56% were *Salmonella enterica*, and ~1% were *E. coli* or *Klebsiella pneumoniae* (**Figure 1D**). Of the bacteria within the Enterococcaceae family, 97% belonged to *Enterococcus faecalis* (3.51% of total bacteria). A significant number of sequences, ~16%, were unclassified, yet many of the pathogens associated with LOS were represented within this combination of bacteria.

### NEC’teria results in predictable, dose-dependent mortality

**Figure 2:**
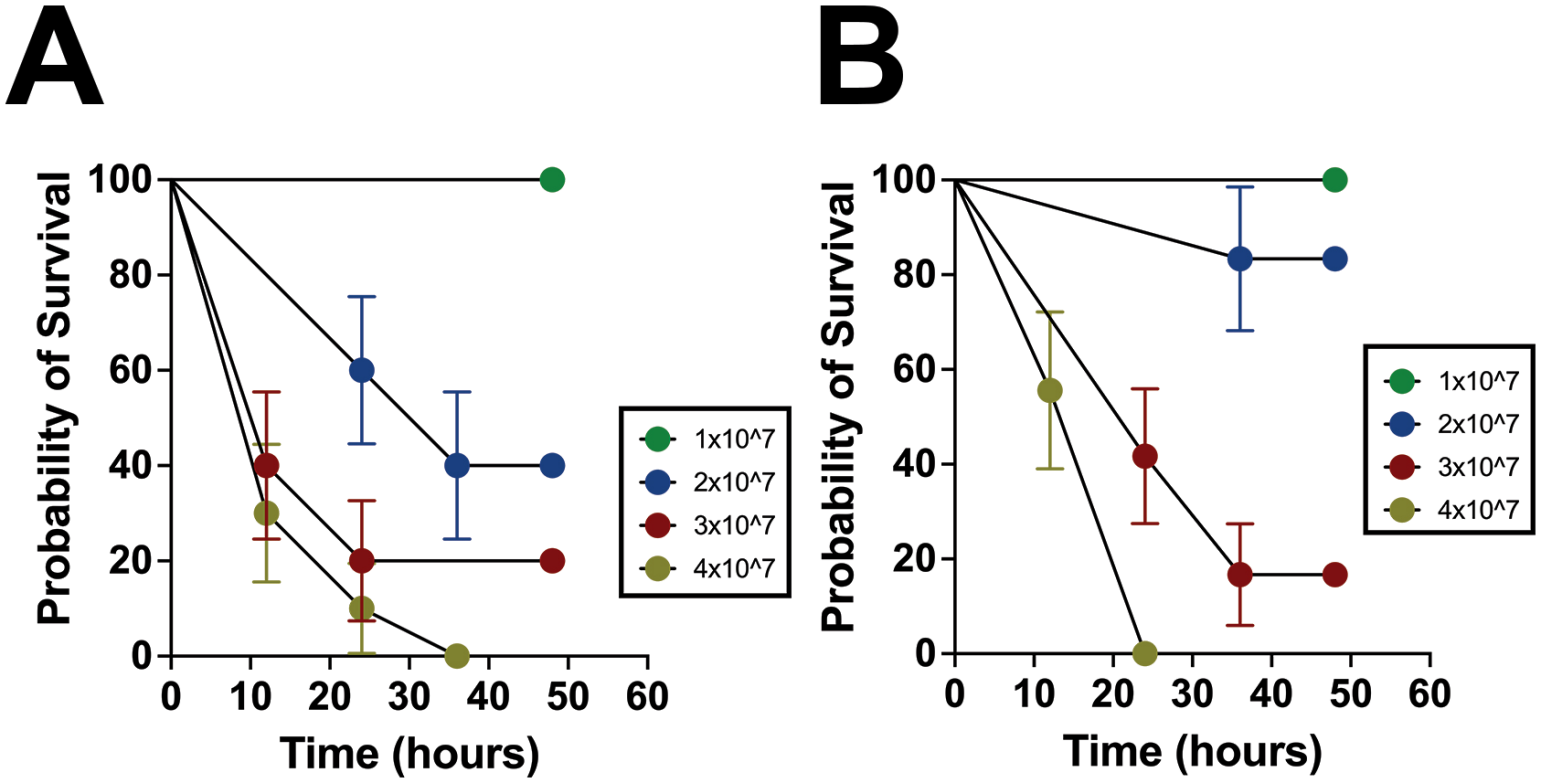
Characterization of survival and inflammation in the pathogenic slurry LOS model. **(A)** P14 – P16 survival curves in concentrations of NEC’teria between 1 x 10^7^ and 4 x 10^7^ through 48 hours following injection. Groups were determined to be significant (p < 0.0001) by log-rank mantel-cox analysis, n = 15 per group. Circles represent the mean with error bars indicating the standard error of the mean. **(B)** P7 mortality in concentrations of NEC’teria 1 x 10^7^ and 4 x 10^7^ through 48 hours following injection. Groups were determined to be significant (p < 0.0001) by log-rank mantel-cox analysis, n = 9 per group. Circles represent the mean with error bars indicating the standard error of the mean.

To model a septic event following spillage of intestinal bacteria into the peritoneal cavity similar to that which would occur after a spontaneous intestinal perforation (SIP) or NEC, P14 – P16 pups were injected intraperitoneally with varying concentrations of NEC’teria to determine survival through 48 hours (**Figure 2A**). Mice injected with the 1 x 10^7^ concentration sustained no mortality, while those that received the 4 x 10^7^ dose experienced 100% mortality by the 36-hour time point. The intermediate doses, 2 x 10^7^ and 3 x 10^7^, resulted in 60% and 80% mortality, respectively, by the 48-hour time point. Resulting in 60% mortality by 12 hours, the 3 x 10^7^ dose was selected as the median lethal dose (LD50) for future 12-hour experiments.

Since both NEC and SIP are gestation- and developmental stage-dependent, to better understand the relationship between bacterial dose and age, the experiment was repeated using the same doses in P7 mice (**Figure 2B**). Similar to the P14 – P16 mice, no mortality was observed within 48 hours in mice receiving the 1 x 10^7^ dose, while the 4 x 10^7^ dose induced complete mortality within 24 hours, earlier than in P14–16 mice. Interestingly, the 2 x 10^7^ dose in P7 mice resulted in 83% survival by the 48-hour time point, compared to only 40% survival in their older counterparts. The 3 x 10^7^ dose in the P7 pups resulted in 83% mortality by the 48-hour time point, similar to the P14 – P16 mice. In the P7 pups, the 3 x 10^7^ dose resulted in roughly 60% mortality by the 24-hour time point, which was 12 hours later than an equivalent mortality percentage in the P14 – P16 pups. Overall, the mortality observed was consistent and predictable within the age groups tested and was relatively consistent between the two age groups as well.

### Mortality, inflammation, and intestinal injury following NEC’teria injection are dependent on live bacteria

**Figure 3:**
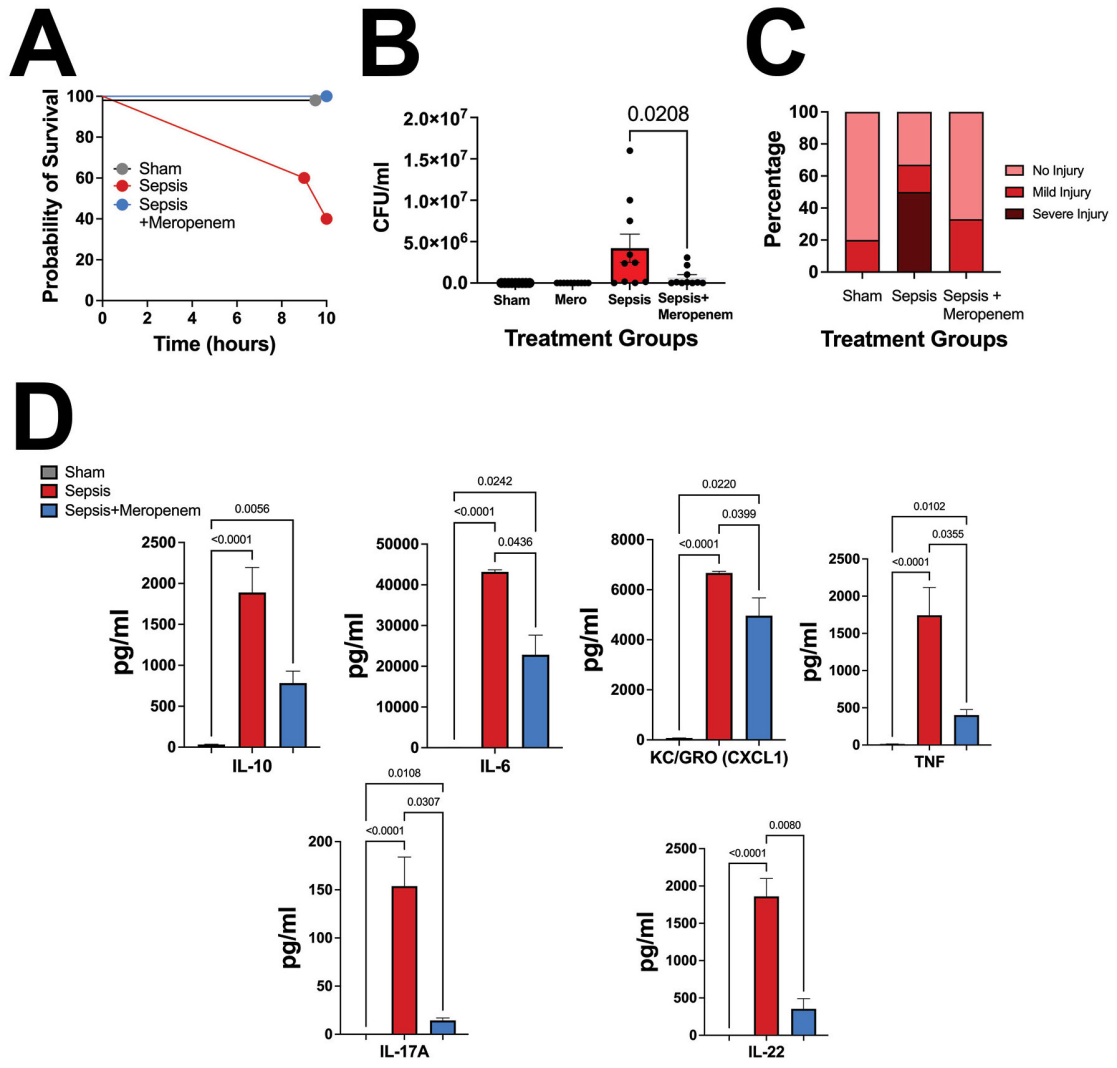
Pathologic slurry sepsis survival and injury are dependent on live bacteria. **(A)** P14 C57Bl/6 animals were treated with NEC’teria, NEC’teria + Meropenem, or sham controls and monitored for survival. Meropenem treatment one hour following LOS induction eliminated all mortality. **(B)** Ten hours following sepsis induction, animals were euthanized, and peritoneal washes were harvested to determine Meropenem effectiveness. Data shown is CFU grown overnight from peritoneal wash harvests. Significance was determined by Sidak’s multiple comparisons test with significance as shown, n ≥ 8 per group. **(C)** Intestines were harvested and scored for injury. No significance was determined between groups, but the sepsis alone group had a trend toward increased injury compared to sham and sepsis + Meropenem groups (n = 9 per group). **(D)** Cytokine comparison of IL-10, IL-6, KC-GRO, TNF, and IL-17A between sham, sepsis-treated, and sepsis + meropenem treated animals. A Dunn’s multiple comparisons test used to determine significance, error bars signify SEM, n = 8 animals per group.

To determine whether the mortality observed was dependent on live bacteria or attributable to another factor, such as systemic immune response, a Meropenem rescue group was developed. Meropenem is a broad-spectrum antibiotic used within the NICU, with intended efficacy towards the bacteria present within NEC’teria (Sabet et al., 2018). Pups that received IP Meropenem without NEC’teria exposure sustained no mortality over a 12-hour period, validating the safety of Meropenem treatment for neonatal mice (**Figure 3A**). Notably, we also observed complete survival in pups that received Meropenem one hour after a 3 x 10^7^ CFU dose of NEC’teria. In contrast, pups that received the same dose of NEC’teria without Meropenem experienced 60% mortality by the 12-hour time point.

To assess inflammation induced by NEC’teria exposure, serum cytokines were quantified 10 hours after initial injection (**Figure 3D**). Significant increases in serum concentrations of IL-6, IL-10, KC-GRO, TNF, and IL-17A were observed in pups exposed to NEC’teria when compared to sham controls. This inflammatory effect appears to be partially attenuated by the administration of the antimicrobial agent, Meropenem, one hour after sepsis induction. In mice that received an injection of NEC’teria, receiving Meropenem significantly decreased serum levels of IL-6, KC-GRO, TNF, and IL-17A compared to pups who received no antibiotics (p values and n as depicted in **Figure 3D**). However, Meropenem administration was not sufficient to return cytokine levels to baseline and was not shown to affect IL-10 response.

Intraperitoneal washes confirmed that sham mice did not face any significant bacterial burden in the peritoneum, regardless of Meropenem administration (**Figure 3B**). Pups that received a NEC’teria injection experienced the highest mean bacterial burden in the peritoneum (4.2x10^6^ CFU), but the addition of Meropenem significantly reduced (p = 0.0208) the mean bacterial burden (6.5x 10^5^ CFU) in the peritoneum.

We next wanted to examine the impact of NEC’teria exposure to intestinal injury scores on a generalized scale (**Figure 3C**). Using a validated immature intestinal injury score developed by our laboratory (Wynn et al., 2016) (Lueschow-Guijosa et al., 2024), sham pups exposed to PBS followed by Meropenem, 20% experienced mild intestinal injury and 80% experienced no intestinal injury. In contrast, pups exposed to NEC’teria alone experienced severe intestinal injury in 50% of animals, while 17% experienced mild injury and 33% remained without injury. Meropenem appeared to ameliorate the damaging effects of NEC’teria on the intestine. Pups that received Meropenem after NEC’teria exposure experienced mild (33%) or no (67%) intestinal injury.

### NEC’teria injection significantly impacts the microbiome

**Figure 4:**
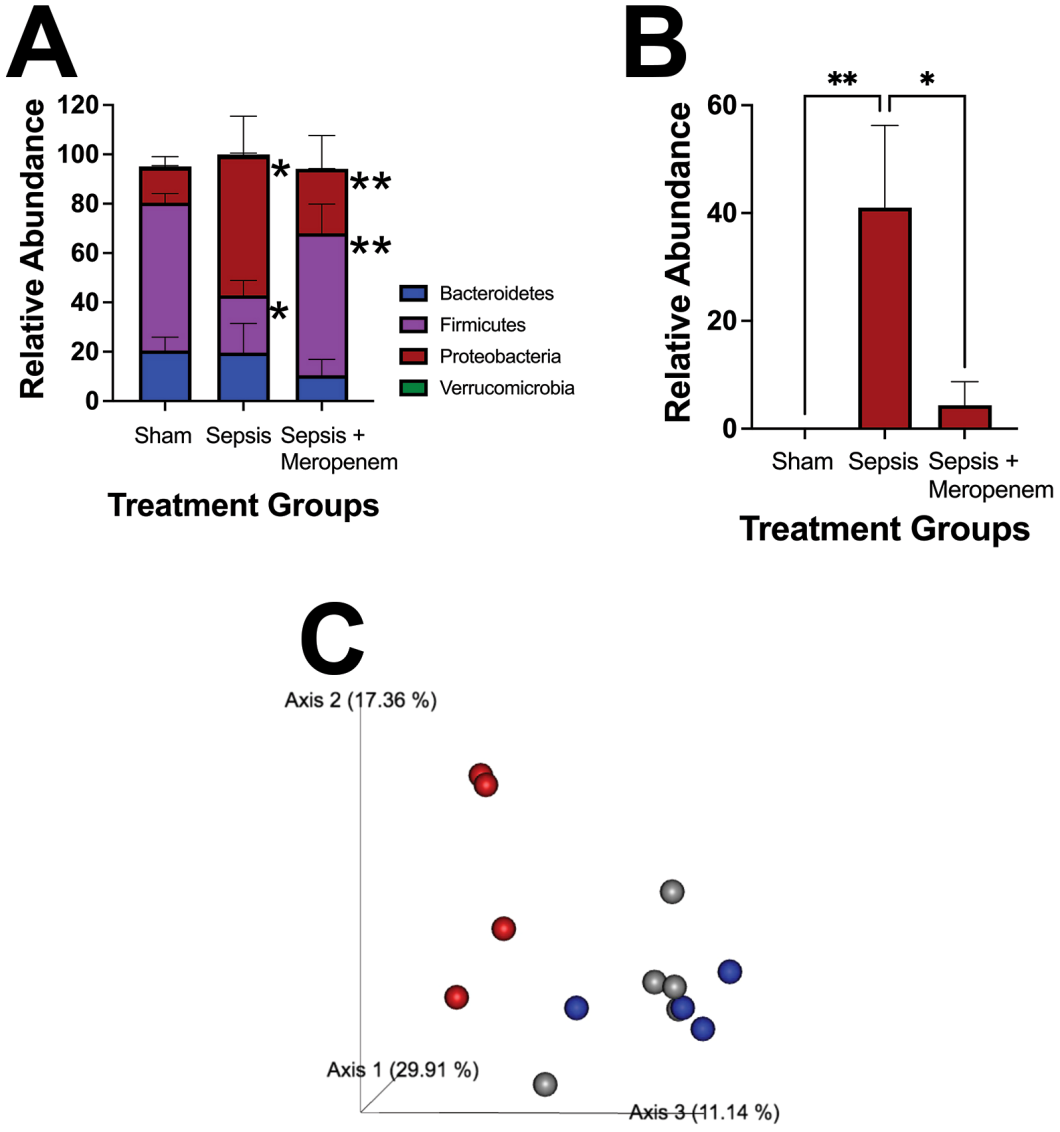
Characterization of pathologic slurry induced sepsis on the cecal microbiome. Induction of LOS via IP injection of NEC’teria induced a significant change in composition of the **(A)** Phylum level microbiome and specifically in **(B)** the Enterobacteriaceae family. N = 5 animals per group. Bars represent the mean values with error bars representing the standard error of the mean. n = 5 for each treatment group. A Two-way ANOVA was used to analyze the relative abundance differences with a Dunnett’s multiple comparisons test used to determine differences between individual groups. Significance is noted as * < 0.05, and ** < 0.001. **(C)** Principal coordinate analysis (PCoA) comparing the beta diversity of the three treatment groups is shown. Gray spheres represent sham mice, red spheres represent sepsis mice, and blue spheres represent sepsis + Meropenem mice. The axes shown represent linear combinations of the original variables to capture maximal variance in data to allow for visualization of similarities and differences.

We next sought to determine if the presence of the NEC’teria in the peritoneal space had an independent effect on intestinal microbiome health. We first examined the cecal microbiome twelve hours following infection. At the phylum level, sepsis exposed mice had a significantly higher relative abundance of Proteobacteria (p = 0.0027) and a significantly lower relative abundance of Firmicutes (p = 0.0099) compared to sham mice (**Figure 4A**). In contrast, mice that were exposed to sepsis and then treated with Meropenem had no difference in either phylum compared to sham. However, sepsis pups treated with Meropenem had significantly higher relative abundance of Firmicutes (p = 0.0168) and significantly lower relative abundance of Proteobacteria (p = 0.0376) compared to the sepsis alone group. At the family level, sepsis exposed mice had significantly higher levels of Enterobacteriaceae (p ≤ 0.04) than both the sham and Meropenem treatment group (**Figure 4B**). Further, when analyzing beta diversity with a principal coordinate analysis (PCoA), the sepsis group clustered distinctly from the sham and Meropenem treated groups which clustered more similarly (**Figure 4C**).

### NEC’teria exposure induced significant changes in local and systemic immune cell expression at four hours

**Figure 5:**
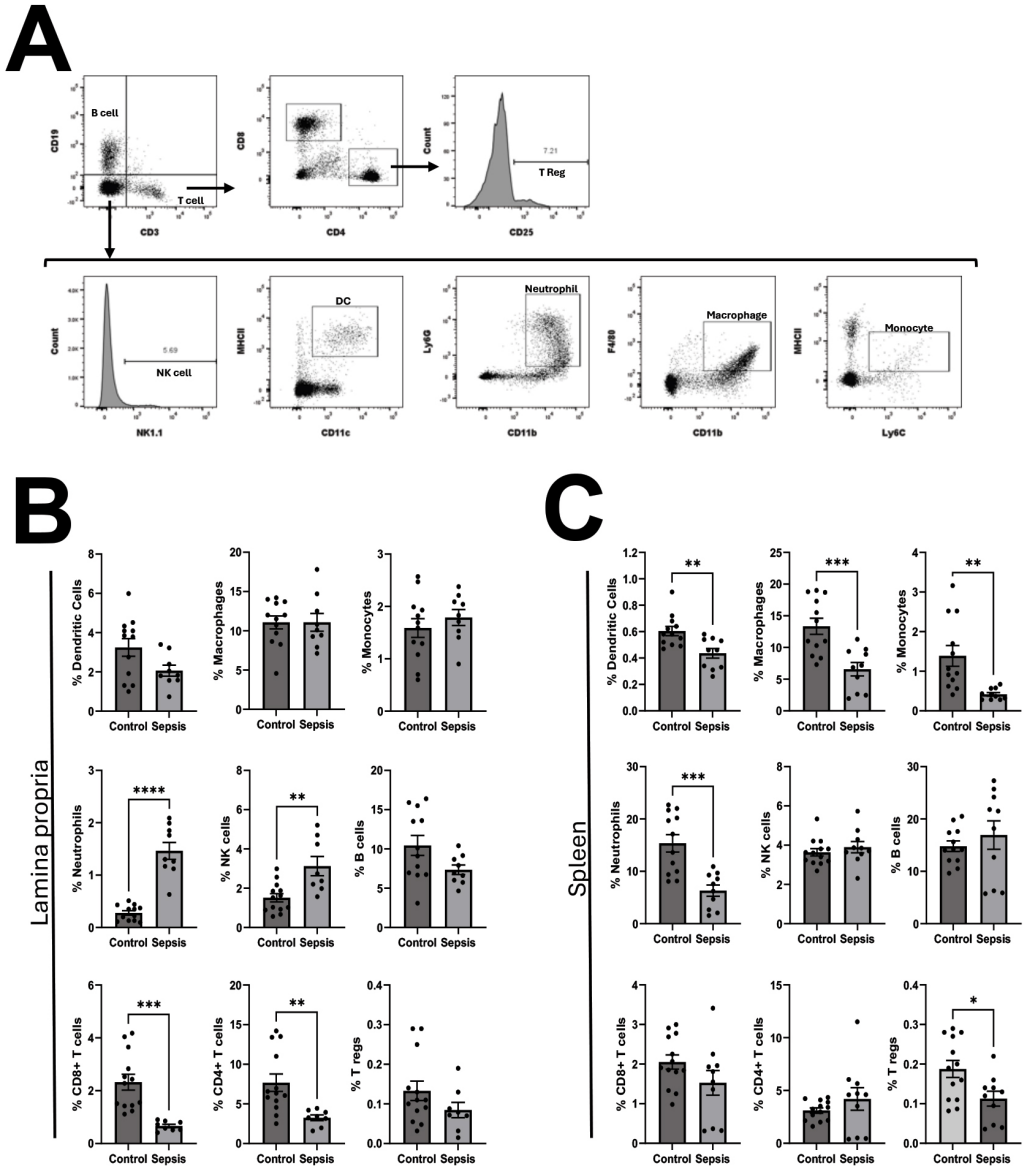
Characterization of pathologic slurry induced sepsis on the relative abundance of leukocytes in the lamina propria and spleen. Four hours following injection, mice were euthanized and single cell suspensions of leukocytes from lamina propria and spleens were processed for flow cytometry as described in methods. **(A)** Representative gating strategy: Single cell leukocytes were first gated by CD3 and CD19. Among CD3+, CD19- cells, CD4 helper T-cells were defined as CD4+, CD8- and CD8 cytotoxic T cells were defined as CD8+, CD4-. Regulatory T cells were identified as CD4+, CD25+. B cells were defined as CD19+, CD3-. Among CD3-,CD19- cells, Natural Killer cells were defined as NK1.1+, Dendritic cells as MHCII+, CD11c+, Neutrophils as Ly6G+, CD11b+, macrophages as F4/80+, CD11b+ and monocytes as MHCII^low^, Ly6C+. The relative expression of leukocyte populations within the **(B)** Lamina Propria (LP) and **(C)** Spleen was compared between control mice and sepsis exposed mice. Comparisons were made by unpaired T tests. Bars represent the mean values with error bars representing the standard error of the mean. Control LP (N = 12), Sepsis LP (N = 9), Control Spleen (N = 12), Sepsis Spleen (N = 10), significance noted as *p < 0.05; **p < 0.01; ***p < 0.001; ****p < 0.0001.

To determine the impact of NEC’teria on immune cell expression both locally in the LP and systemically, expression of various innate and adaptive immune cell types was determined by flow cytometry from the LP and spleen four hours following injection of bacteria (**Figure 5**). Among sepsis exposed mice, the expression of Ly6G+, CD11b+ Neutrophils and NK1.1+ NK cells in the LP was significantly increased compared to controls (Neutrophils p < 0.0001; NK cells p = 0.0025) (**Figure 5B**). The expression of CD4+ and CD8+ T cells in the LP was significantly decreased among sepsis exposed mice compared to controls (CD4+ T cells p = 0.006; CD8+ T cells p = 0.0004) (**Figure 5B**). There were no significant differences in LP expression of MHCII+, CD11c+ DCs; F4/80+, CD11b+ Macrophages; MHCII^low^, Ly6C+ Monocytes; CD19+ B cells; or CD4+, CD25+T regs between sepsis exposed and control mice at four hours (**Figure 5B**). Within the spleen, sepsis exposed mice had decreased expression compared to control mice of DCs (p = 0.004), Macrophages (p=0.0008), Monocytes (p = 0.003), Neutrophils (p = 0.0003) and T regs (p = 0.02)(**Figure 5C**). There was no significant difference in the expression of CD4+T cells, CD8+ T cells, B cells or NK cells from spleens of sepsis exposed mice versus control mice (**Figure 5C**).

## Discussion

Our pathogenic sepsis model uses a modification of Wynn’s cecal slurry model to simulate late-onset sepsis following a NEC or SIP event where intestinal injury/perforation causes bacteria from the lumen of the intestine to spill into the peritoneal cavity. This adapted model results in consistent and predictable mortality, elevated inflammatory cytokines, and modulation of the innate immune response that is dependent on live bacteria. Using NEC’teria instead of a cecal bacteria slurry from healthy adult mice, as described in previous models, may offer more relevance to neonatal sepsis, as the bacteria is known to be pathogenic and specific to a neonate. We also expect our model to be highly reproducible between institutions, due to the standardized bacterial dose and characterized stock solution. One limitation of the CS model is that despite efforts to reduce variation, different CS batches have varying composition (**Figure 1**) and pathogenicity, causing variable sepsis-induced mortality (Brook et al., 2019; Davenport et al., 2022). By nature of the protocol, each batch of CS is finite and not cultured or maintained. Modifications by Starr et al. allow for long-term storage of a stock to reduce, but not fix, this problem (Starr et al., 2014). Utilizing a stock of NEC’teria that has been cultured and used by other research institutions allows for higher reproducibility and potentially mitigates variation arising from differences in cecal microbiomes due to donor mouse supplier, diet, age, and vivarium.

Our pathologic sepsis model was successful in recapitulating a disease phenotype comparable to what is observed in LOS in human preterm infants. First, the cytokines IL-6, TNF, and IL-10 have been found in many studies to be significantly elevated in human infants experiencing LOS (Ye et al., 2017) (Shane et al., 2017), as well as other mouse models of neonatal sepsis (Flierl et al., 2008) (Wynn et al., 2007). Our model saw similar increases in these cytokines, as well as increases in KC-GRO and IL-17A. In our study, while neutrophil and NK cell expression was relatively increased, there was a decrease in expression of T cells and B cells in sepsis-exposed mice both in the LP and spleen. This is consistent with current literature using the CS model of sepsis induction, with increased neutrophils, decreased T and B cell expression, and downregulation in leukocyte expansion and recruitment gene signaling and decreased live immune cell counts in sepsis exposed neonatal mice (Gentile et al., 2014; Young et al., 2017). In human neonates, leukopenia, predominantly from depressed lymphocyte counts, as well as a predominantly innate immune response has been well described in neonatal sepsis (Adane et al., 2022; Das et al., 2024). Interestingly, Wynn et al. saw increased splenic CD4+, CD8+, and Treg expression at 24 hours after CS injection, but no differences at 12 hours, suggesting that the neonatal adaptive arm of the immune response develops at a later time point which our current study may not capture prior to mortality without meropenem rescue. The ideal timing for evaluating cellular expression changes in sepsis is dependent on the relative pathogenicity of the model, and the branch of the immune system of interest. While our model has significant early mortality, it more accurately approximates survivability times seen in human LOS without antibiotic treatment and may provide a more clinically relevant model of early innate immune responses to highly pathogenic exposures.

LOS results in damage to the gastrointestinal tract, which presents in infants as abdominal distension, vomiting, diarrhea, as well as hepatomegaly (Shane et al., 2017). In our mice, we observed severe injury to the intestine in septic mice, facilitated by NEC’teria, but partially alleviated by early antibiotic treatment with Meropenem. While there were no significant differences between groups, the distribution of injury in sepsis pups that received Meropenem is more similar to sham controls than to other sepsis mice, suggesting that NEC’teria exposure may impact intestinal injury, but only if the bacteria are alive. We additionally observed significant increases in Enterobacteriaceae species in the cecal microbiome of sepsis exposed pups compared to the sham controls and sepsis + Meropenem pups. The recovery of bacteria by IP wash gave confirmation of bacterial colonization of the peritoneum, as may be seen clinically in intra-abdominal neonatal sepsis following NEC (Mollitt et al., 1988).

One of the limitations of the cecal slurry model described by Wynn et al. is the use of healthy adult donor mouse cecal slurries, which may or may not have pathogens relevant to LOS. Choosing an appropriate bacterial composition to the population being studied is critical, as it has significant implications for the specific immune response launched (Davenport et al., 2022). The bacterial composition of the gastrointestinal tracts of both mice and humans are highly variable between adults and neonates as the neonatal gut microbiome is highly influenced by microbes *in utero* (Collado et al., 2016) (Jiménez et al., 2008) (DiGiulio et al., 2008). Further, infants born prematurely face additional factors that alter microbiome composition, such as extended hospital stays, formula feeds, and antibiotics (Penders et al., 2006) (Korpela et al., 2018) (Ihekweazu and Versalovic, 2018). The bacteria causing sepsis in our pathogenic sepsis model consist of known pathogens in families that dominate the microbiome of preterm infants, including members of the Enterobacteriaceae family (such as *E. cloacae*, *E. coli*, *K. pneumoniae*, *C. sakazakii*, and *S. enterica)* as well as Enterococcaceae family members, like *E. faecalis (* Ihekweazu and Versalovic, 2018 *)*. The majority of the bacteria we found were gram-negative, which are associated with the highest rates of sepsis related morbidity and mortality and therefore understanding the specific immune response to these bacteria in neonatal LOS are of critical importance (Fleiss et al., 2021) (Tsai et al., 2014) (Dong et al., 2019) (Stoll et al., 2002). Few accounts exist about the composition of CS models, but those that do show a majority of Firmicutes and considerably less Proteobacteria than our NEC’teria slurry (Karner et al., 2020) (Bongers et al., 2024) (Davenport et al., 2022). Our data support these findings as both cohorts of mice received from Jackson Laboratories had a diverse bacterial composition that consisted of common non-pathogenic strains. In both cohorts, there was approximately 40% Firmicutes, 10-20% Bacteroidetes, and miniscule amounts of Proteobacteria. Therefore, it is likely NEC’teria adheres more closely with the recommendation by the International Expert Consensus for Pre-Clinical Sepsis Studies that microorganisms used in animal models should preferentially reflect those commonly found in human sepsis (Osuchowski et al., 2018). However, while our model provides a clinically relevant model for LOS and sepsis highly associated with NEC, it may be less relevant in EOS studies (Clyman et al., 2020). Similarly, it may not accurately model meconium peritonitis where sterile meconium enters the abdominal cavity following an intestinal perforation before significant establishment of the intestinal microbiome. A recent murine work showed that IP injection from a stock slurry of sterile human meconium can induce dose-dependent mortality and increased inflammation in four-day-old mouse pups (Ashina et al., 2024). Therefore, choosing the inducing flora should be based on the questions being asked in the study and the disease being modeled.

Overall, our pathogenic sepsis modification to the cecal slurry neonatal sepsis model resulted in consistent sepsis-related mortality and phenotypic changes in neonatal mouse pups that resembled the changes that occur in human preterm infants who develop LOS. We hope these preliminary results will encourage more research into the characterization of this model to assess both its strengths and limitations in recapitulating LOS. Eventually, we hope the use of this model may prove valuable in learning more about the neonatal response to sepsis and in testing potential interventions.

## Data Availability

The datasets presented in this study can be found in online repositories. The names of the repository/repositories and accession number(s) can be found below: https://datadryad.org/stash, https://doi.org/10.5061/dryad.0p2ngf2bv.
